# International relevance of two measures of awareness of age-related change (AARC)

**DOI:** 10.1186/s12877-020-01767-6

**Published:** 2020-09-21

**Authors:** Serena Sabatini, Obioha C. Ukoumunne, Clive Ballard, Allyson Brothers, Roman Kaspar, Rachel Collins, Sarang Kim, Anne Corbett, Dag Aarsland, Adam Hampshire, Helen Brooker, Linda Clare

**Affiliations:** 1 College of Medicine and Health, REACH, University of Exeter, South Cloisters, St Luke’s Campus, Exeter, EX12LU UK; 2 NIHR ARC South West Peninsula (PenARC), University of Exeter, Exeter, UK; 3 College of Health and Human Sciences, Colorado State University, Fort Collins, Colorado USA; 4 Cologne Center for Ethics, Rights, Economics, and Social Sciences of Health, University of Cologne, Cologne, Germany; 5 Wicking Dementia Research & Education Centre, University of Tasmania, Hobart, Australia; 6 Department of Medicine, Imperial College London, London, UK; 7 Department of Brain Sciences, Imperial College London, London, UK

**Keywords:** Subjective aging, Self-perceptions of aging, AARC-10 SF, Cognitive functioning

## Abstract

**Background:**

A questionnaire assessing awareness of positive and negative age-related changes (AARC gains and losses) was developed in the US and Germany. We validated the short form of the measure (AARC-10 SF) and the cognitive functioning subscale from the 50-item version of the AARC (AARC-50) questionnaire in the UK population aged 50 and over.

**Methods:**

Data from 9410 participants (Mean (SD) age = 65.9 (7.1)) in the PROTECT cohort were used to explore and confirm the psychometric properties of the AARC measures including: validity of the factor structure; reliability; measurement invariance across men and women, individuals with and without a university degree, and in middle age, early old age, and advanced old age; and convergent validity with measures of self-perception of aging and mental, physical, and cognitive health. We explored the relationship between demographic variables (age, sex, marital status, employment, and university education) and AARC.

**Results:**

We confirmed the two-factor structure (gains and losses) of the AARC-10 SF and the AARC-50 cognitive functioning subscale. Both scales showed good reliability and good convergent validity for AARC losses, but weak convergent validity for AARC gains. For both scales metric invariance was held for the two subgroups defined by education level and age. For the AARC-50 subscale, but not for the AARC-10 SF, strong invariance was also held for the two subgroups defined by sex. Age, sex, marital status, employment, and university education predicted AARC gains and losses.

**Conclusions:**

The AARC-10 SF and AARC-50 cognitive functioning subscale identify UK individuals who perceive age-related changes in their mental, physical, and cognitive health.

## Background

Awareness of age-related change (AARC) is a useful concept that predicts a variety of health-related outcomes such as depression and psychological and physical well-being [1, 2] and could be used to motivate engagement in healthy behaviors such as physical activity [3, 4]. AARC refers to “a person’s state of awareness that his or her behavior, level of performance, or way of experiencing life has changed as a consequence of having grown older” [5], p. 342. AARC reflects the observation that individuals’ experiences of aging may vary across five life and behavioral domains including health and physical functioning, cognitive functioning, interpersonal relationships, socio-cognitive and socio-emotional functioning, and lifestyle/engagement. As the association between cognitive complaints and cognitive performance is well-reported in the empirical literature (e.g., [6, 7]), among the five AARC behavioral domains, the cognitive functioning domain is potentially useful for detecting early stages of cognitive decline. AARC captures awareness of both positive (AARC gains) and negative (AARC losses) age-related changes and acknowledges that AARC gains and losses can coexist, even in the same behavioral domain [5].

A questionnaire assessing AARC exists in three published versions of differing length and across two languages (English and German). The 50-item version [8] and short 10-item version (AARC-10 SF; [1]) are available in English. In the full 50-item version, out of the 50 items, half represent perceived gains and half perceived losses. There are five gain- and loss-related items representing each of the five theorized domains. The AARC-50 questionnaire has been shown to have good reliability in a sample of US residents aged between 42 and 98 years old [8], with Cronbach’s alpha (*α*) coefficients ranging from .73 to .89 across all ten subscales.

The 10-item version of the AARC questionnaire is made up of selected items from the AARC 50-item version. Reliability of the AARC-10 SF is good among US and German residents aged 40 and over (Cronbach’s *α* coefficients ranging from .49 to .75 across subscales) [1]. A 20-item modified version of the AARC questionnaire adapted for daily use also exists [9]. The 20 items have been selected from the 50-item version of the AARC questionnaire. However, each item stem, instead of asking participants to reflect on their increasing age (“With my increasing age…”), invites participants to reflect on their awareness of aging in that specific day (“With my awareness of aging today…”). Psychometric properties of the AARC 20-item version have never been explored.

AARC may be associated with cognitive functioning (e.g., [10]). As the AARC-10 SF includes only two items assessing AARC gains and AARC losses respectively in the cognitive domain, the full 10-item subscale assessing AARC gains taken from the AARC 50-item version of the questionnaire makes it possible to more accurately explore the potential associations of AARC in the cognitive domain with other indicators of cognitive functioning. The AARC-10 SF and the AARC-50 cognitive functioning subscale may be particularly important when thinking about new ways of preventing poor mental and physical health and cognitive decline.

The AARC-10 SF [1] and the AARC-50 cognitive functioning subscale [8] are suitable to be used in long surveys or as screening tools to identify those people at greater risk of poor mental and physical health and/or cognitive decline [1, 10]. In order to use these measures in the UK, due to potential cross-cultural differences in AARC, it is important to first explore their psychometric properties in the UK population aged 50 and over [11]. German participants, for example, report fewer AARC gains, assessed with the AARC-10 SF, than US participants [12]. Studying individuals aged 50 years and above is considered appropriate as people in this age-group are old enough to be likely to experience AARC. Individuals aged 50 years have previously reported experiencing many age-related changes (e.g., [13–15]) and shown concern about their physical health [16].

Amongst psychometric properties, it is important to test measurement invariance to explore whether the AARC concept is interpreted consistently in the same way across different population groups (for example, defined by sex, level of education, or age) [12, 17]. Estimated reliable comparisons of AARC scores among groups can, therefore, be potentially calculated [18–21]. Regarding the AARC questionnaires, measurement invariance has so far been tested only for the AARC-10 SF in relation to different age groups [1].

Other measures of the subjective experience of aging such as felt age, which reflects how old individuals feel they are [22, 23], and attitudes towards own aging (ATOA), which capture individuals’ evaluations of the changes taking place in their lives as they age [24], are suitable measures to capture the way in which individuals experience aging, albeit in a more holistic manner compared to AARC [3]. These constructs, therefore, were used as part of the exploration of convergent validity of existing AARC questionnaires.

Moreover, as the AARC-10 SF covers awareness of changes in several behaviors and life domains including socio-emotional, physical, and cognitive functioning, investigating the associations of the AARC-10 SF with indicators of mental, physical, and cognitive health provides information about the construct validity of the AARC-10 SF. As part of the US and German validation of the AARC-10 SF it has been found that AARC is associated with indicators of mental and physical health including psychological well-being, satisfaction with life, depressive symptoms, and functional and perceived health [1]. However, despite age playing a role in levels of AARC [12, 25], construct validity of the AARC-10 SF has not been explored in individuals younger than 70 years. Moreover, research shows that higher levels of AARC losses (measured with a 20-item version of the AARC questionnaire) are associated with more negative affect [9, 25] which is a key component of anxiety. As common difficulties among older individuals, such as poverty and diminished life expectations, are risk factors for anxiety [26], the association between anxiety and AARC should also be considered when exploring convergent validity of the AARC-10 SF. Finally, construct validity of the AARC-50 cognitive functioning subscale [8] in relation to objective or subjective cognitive assessments has never been explored.

Existing research in the US and Germany, conducted by using the 50-item and 20-item versions of the AARC questionnaire, suggests that on average individuals who are older, less well-educated, and/or female have higher levels of both AARC gains and AARC losses [12, 17]. These findings are in line with research conducted on other constructs that, similarly to AARC, also capture individuals’ self-perceptions of aging [23, 27, 28]. Individuals who report a higher socioeconomic status tend to experience more AARC gains and fewer AARC losses (assessed with the 20-item version of the AARC questionnaire) than those with a lower socioeconomic status [17]. However, the role of demographic variables in the UK population is unexplored. Moreover, other demographic variables such as marital status and employment status have never been explored in relation to AARC gains and losses even though existing literature suggests that they may influence individuals’ perceptions of aging [29].

This study aims to: (a) confirm the two-factor structure (one factor for each of gains and losses) and internal consistency of the AARC-10 SF [1] and the AARC-50 cognitive functioning subscale [8]; (b) explore measurement invariance for the AARC-10 SF and for the AARC-50 cognitive functioning subscale among subgroups defined by sex, education level, and age; (c) explore construct validity of the AARC-10 SF and the AARC-50 cognitive functioning subscale by quantifying the associations of the AARC-10 SF with assessments of subjective aging experiences, physical, mental, and cognitive health and of the AARC-50 cognitive functioning subscale with assessments of subjective aging experiences and cognitive health; and (d) explore whether demographic variables predict scores on the AARC-10 SF and AARC-50 cognitive subscale gains and losses.

## Methods

### Study design and participants

The study was based on analyses of cross-sectional data collected through the ongoing PROTECT [30] study in 2019. PROTECT is a 25-year longitudinal study launched in 2014 that assesses participants every year on measures of physical, mental, and cognitive health, lifestyle, and perceptions of aging through an online platform.

Individuals are eligible to participate in the PROTECT study if they are UK residents, English speakers, aged 50 years and over, have access to a computer and internet, and do not have a clinical diagnosis of dementia at the point of recruitment. Participants were recruited through national publicity and via existing cohorts of older adults. Potential participants enrolled through the PROTECT study website, downloaded the study information sheet, and provided consent online.

The PROTECT study has ethical approval from the London Bridge NHS Research Ethics Committee and Health Research Authority (Ref: 13/LO/1578). Ethical approval for the data analyses was sought through the ethics committee at the University of Exeter, School of Psychology (Application ID: eCLESPsy000603 v1.0).

Between 1st January 2019 and 31st March 2019, 14,797 participants took part in the PROTECT annual assessment. Among these, 9410 participants completed the AARC questionnaires and were therefore included in the present study (mean (SD; range) age: 65.9 (7.1; 51–95) years). Only 0.4% of participants reported having been diagnosed with mild cognitive impairment. We estimated that a further 1.2% of participants had mild cognitive impairment (as they scored 1.5 SDs below the mean study sample score in two or more cognitive tasks). Those participants that we identified as having mild cognitive impairment were kept in the analyses. However, participants with higher levels of AARC losses on the AARC-10 SF and on the AARC-50 cognitive functioning subscale had poorer scores on the four objective cognitive tasks, indicating that participants were aware of their cognitive abilities (see supplementary material Table 1) and hence their answers to the AARC-10 SF and the AARC-50 cognitive functioning subscale can be deemed accurate.

The majority of study participants was of white ethnicity (98.5% of participants), married (79.1% of participants), completed a university education (75.8% of participants) and not retired (42.6% of participants). Demographic characteristics for the study sample are reported in Table 1. Means and standard deviations stratified by age, sex, and education level for AARC gains and losses assessed both with the AARC-10 SF and the AARC-50 cognitive functioning subscale are reported in Tables 2 and 3.

**Table 1:** Demographic characteristics of the study sample (*N* = 9410)

Characteristics	Statistic
Age (years), M (SD)	65.9 (7.1)
Range	51–95
Sex (Women %)	79.9
Ethnicity (%)	
White	98.5
Mixed	0.5
Asian	0.6
Black	0.1
Other ethnic groups	0.3
Marital status (%)	
Married/ civil partnership/ co-habiting	79.1
Widowed/ separated/ divorced/ single	20.9
University education (Yes %)	75.8
Current employment (Yes %)	42.6

N = 9410

University education was operationalized as a dichotomous variable. No university education included those participants that concluded secondary education or post-secondary education. University education included those participants that concluded vocational qualification, undergraduate degree, post-graduate degree, or doctorate. Secondary education = GCSE or O-levels. Post-secondary education = College, A-levels, NVQ3 or below, or similar. Vocational qualification = Diploma, certificate, BTEC, NVQ 4 and above, or similar. Undergraduate degree = BA or BSc, or similar. Post-graduate degree = MA, MSc, or similar. Doctorate = PhD

**Table 2:** Levels of AARC gains and losses stratified by age

Age											
	Class 1: 50 to 65 (*N* = 4929)	Class 2: 66 to 75 (*N* = 3758)	Class 3: 76 and over (*N* = 723)	Class 1 vs 2		Class 1 vs 3		Class 2 vs 3		*F statistic (df)*	*p*-value
	Mean (SD)	Mean (SD)	Mean (SD)	Mean difference	[95% CI]	Mean difference	[95% CI]	Mean difference	[95% CI]		
AARC-10 SF gains	18.1 (3.9)	17.8 (3.9)	17.4 (3.7)	−0.3	[−0.5, 0.1]	−0.7	[−1.0, − 0.3]	−0.4	[− 0.8, − 0.1]	12.1 (2)	<.0001
AARC-10 SF losses	9.4 (3.2)	10.2 (3.1)	12.1 (3.8)	0.8	[0.6, 0.9]	2.7	[2.4, 3.0]	1.9	[1.7, 2.2]	243.4 (2)	<.0001
AARC-50 cognitive functioning gains	14.3 (4.4)	13.6 (4.4)	13.3 (4.4)	−0.7	[−0.9, 0.5]	−1.0	[−1.4, − 0.6]	−0.3	[− 0.7, 0.1]	34.5 (2)	<.0001
AARC-50 cognitive functioning losses	9.8 (3.7)	10.4 (3.5)	12.0 (4.1)	0.6	[0.4, 0.8]	2.2	[1.9, 2.5]	1.6	[1.2, 1.9]	123.0 (2)	<.0001

Total sample size (N) = 9410

**Table 3:** Levels of AARC gains and losses stratified by sex and educational level

Sex				
	Women (*N* = 7334)	Men (*N* = 2076)		
	Mean (SD)	Mean (SD)	*t-statistics (df)*	*p*-value
AARC-10 SF gains	18.2 (3.8)	16.9 (4.0)	−14.2203 (9408)	<.0001
AARC-10 SF losses	9.7 (3.2)	10.5 (3.5)	9.2481 (9408)	<.0001
AARC-50 cognitive functioning gains	14.2 (4.4)	13.0 (4.4)	−11.6155 (9408)	<.0001
AARC-50 cognitive functioning losses	10.0 (3.6)	10.9 (3.9)	9.6811 (9408)	<.0001
University education				
	No university education (*N* = 2369)	Completed university education (*N* = 7041)		
	Mean (SD)	Mean (SD)	*t-statistics (df)*	*p*-value
AARC-10 SF gains	18.1 (3.9)	17.9 (3.9)	2.5758 (9408)	=.01
AARC-10 SF losses	10.2 (3.5)	9.8 (3.2)	5.7887 (9408)	<.0001
AARC-50 cognitive functioning gains	14.4 (4.5)	13.8 (4.4)	5.3472 (9408)	<.0001
AARC-50 cognitive functioning losses	10.5 (3.8)	10.1 (3.6)	6.0893 (9408)	<.0001

Total sample size (N) = 9410

University education was operationalized as a dichotomous variable. No university education included those participants that concluded secondary education or post-secondary education. University education included those participants that concluded vocational qualification, undergraduate degree, post-graduate degree, or doctorate. Secondary education = GCSE or O-levels. Post-secondary education = College, A-levels, NVQ3 or below, or similar. Vocational qualification = Diploma, certificate, BTEC, NVQ 4 and above, or similar. Undergraduate degree = BA or BSc, or similar. Post-graduate degree = MA, MSc, or similar. Doctorate = PhD

A high proportion of participants perceived their health as good (54.1%) or excellent (30.8%). On average participants did not report functional difficulties (IADL mean (SD) score = 0.16 (0.77)). Participants had minimal levels of current depressive (mean (SD) = 11.5 (3.0)) and anxiety symptoms (mean (SD) = 9.3 (8.5)), and low levels of both lifetime depressive symptoms (mean (SD) = 2.7 (3.3)) and lifetime anxiety symptoms (mean (SD) = 1.0 (2.1)).

Compared to those who did not complete the AARC questionnaires (*N* = 5387), the study sample included a larger proportion of women (79.9% versus 71.3%) and participants who were better educated (75.8% versus 70.8%), and a lower proportion of individuals who were employed (42.6% versus 54.7%).

### Instruments

Measures assessing felt age, ATOA, mental and physical health, and objective cognitive functioning were used to explore construct validity for the AARC-10 SF. Measures assessing felt age, ATOA, and objective, self-reported, and informant-reported assessments of cognitive functioning were used to explore construct validity for the AARC-50 cognitive functioning subscale. Demographic variables (age, sex, marital status, employment, and university education) were assessed to explore their relationships with levels of AARC gains and losses assessed both with the AARC-10 SF and with the AARC-50 cognitive functioning subscale.

### Demographic variables

Participants provided demographic information through the PROTECT platform at baseline through an online assessment adapted from Office of National Statistics measures, which included data on age, sex, ethnic origin, marital status, employment, and university education. Ethnicity included the following categories: white, mixed (included white and black Carribean, white and black African, white and Asian, any other mixed multiple ethnic background), Asian, black, or other ethnic groups. Marital status was used as a dichotomous variable (individuals who were married, in a civil partnership, or co-habiting were grouped together versus individuals who were unmarried, divorced, separated, or widowed). Employment status was used as a dichotomous variable (employed versus not employed). University education was used as a dichotomous variable (university education versus no university education). Individuals without a university education were those participants that had completed secondary education (GCSE/O levels) or post-secondary education (college, A-levels, NVQ3, or below). Individuals with a university education were those participants that had completed vocational qualifications (diploma, certificate, BTEC, NVQ4, and above), undergraduate degrees (e.g., BA, BSc), post-graduate degrees (e.g., MA, MSc), or doctorates (PhD).

### Awareness of age-related change (AARC)

#### AARC-10 SF

The AARC-10 SF [1] is a brief tool for capturing perceived age-related gains (AARC gains) and losses (AARC losses). It contains ten items, five assessing AARC gains and five assessing AARC losses. Each of these five items assesses a different AARC behavioral domain (health and physical functioning, cognitive functioning, interpersonal relationships, socio-cognitive and socio-emotional functioning, and lifestyle/engagement). All ten items start with the same stem “With my increasing age, I realize that…”. An example of an item capturing AARC gains is “…I appreciate relationships and people much more”, while an example of an item capturing AARC losses is “…I have less energy”. Respondents rate how much each item applies to them on a five-point Likert scale (1 = “not at all”, 2 = “a little bit”, 3 = “moderately”, 4 = “quite a bit”, and 5 = “very much”). Scores can be obtained for the AARC gains and AARC losses subscales by summing items that fall into the respective scales. Scales scores range from a minimum of five to a maximum of 25 with higher scores indicating higher levels of awareness of age-related change.

#### AARC-50 cognitive functioning subscale

The cognitive functioning subscale of the AARC-50 questionnaire [8] includes ten items, five assessing AARC gains and five assessing AARC losses. An example item capturing AARC gains in the cognitive domain is “With my increasing age, I realize that I have become wiser”, while an item capturing losses is “With my increasing age, I realize that I am more forgetful”. Respondents rate how much each item applies to them on a five-point Likert scale (1 = “not at all”, 2 = “a little bit”, 3 = “moderately”, 4 = “quite a bit”, and 5 = “very much”). Scores on the AARC- cognitive functioning gains and AARC- cognitive functioning losses subscales are obtained by summing items that fall into the respective subscales. Subscales scores range from a minimum of five to a maximum of 25 and higher scores indicate higher levels of awareness of age-related change in the cognitive domain.

### Attitudes toward own aging (ATOA)

The ATOA scale is a valid and reliable five-item scale assessing participants’ attitudes toward their own aging taken from the Philadelphia Geriatric Center Morale Scale [24]. For each statement respondents are asked to make temporal comparisons about changes in energy level, perceived usefulness, happiness, and quality of life and to respond on a binary response set (better versus worse, yes versus no). An example item is “Things keep getting worse as I get older”. A proportion-based score can be obtained by summing the participant’s item scores and by dividing it by the number of responses, with a score of one indicating that positive attitudes are implied in all answers and a score of zero indicating that a negative response is implied in all answers.

### Felt age

Felt age was assessed with a single-item question (adapted from the National Survey of Midlife development in the United States; MIDUS [23]) asking participants to write the age (in years) that they feel most of the time. A proportional discrepancy score was calculated by subtracting the participants’ felt age from their chronological age, and by dividing this difference score by participants’ chronological age. A positive value indicates a youthful felt age, whereas a negative value indicates an older felt age.

### Cognitive functioning – objective assessment

Cognitive functioning was measured with the PROTECT Cognitive Test Battery [31–33] which includes four tests: (1) the Grammatical Reasoning task assesses verbal reasoning [34]; (2) the Digit Span task [35] assesses verbal working memory; (3) the Self-ordered Search task measures spatial working memory [36]; and (4) the Paired Associate Learning task [37] assesses visual episodic memory.

For each task a summary score can be obtained by subtracting the number of errors from the number of correct answers. Hence for each task a higher score indicates a better performance. For digit span the summary score can range from 0 to 20. For paired associate learning the summary score can range from 0 to 16. For verbal reasoning the summary score is also obtained by subtracting the number of errors from the number of correct answers, but the score has no set upper or lower limit as the participants can attempt as many trials as they can manage within a specific timeframe. Finally, the summary score for the self-ordered search task can range from 0 to 20.

### Cognitive functioning - informant rating and self-rating

The Informant Questionnaire on Cognitive Decline in the Elderly short form (IQCODE [38, 39]) was administered to an informant close to the participant. The IQCODE is a valid and reliable 16-item questionnaire that asks respondents to rate the cognitive change of someone close to them over the last 10 years. Items describe both cognitive improvement and cognitive decline (an example item is “Remembering things that have happened recently”) and can be answered on a five-point scale (1 = “much improved”, 2 = “a bit improved”, 3 = “not much change”, 4 = “a bit worse”, and 5 = “much worse”). The final score is the mean of the item scores. A parallel version of the IQCODE was administered to the participant (IQCODE - Self [38]).

### Mental health

#### Patient health Questionnaire-9

The Patient Health Questionnaire-9 (PHQ-9 [40]) is a valid and reliable nine-item scale capturing depressive symptoms over the previous 2 weeks. It is based directly on the diagnostic criteria for major depressive disorder described in the Diagnostic and Statistical Manual Fourth Edition (DSM IV [41]). Respondents are asked to indicate how frequently they experience each symptom on a four-point Likert scale (1 = “not at all”, 2 = “several days”, 3 = “more than half the days”, and 4 = “nearly every day”). The total score is the sum of the item scores and can range from 9 to 36.

#### Composite international diagnostic interview-short form

The Composite International Diagnostic Interview-Short Form (CIDI-SF [42]) is a reliable and valid measure for assessing lifetime symptoms of depression and anxiety. Nine items assess depressive symptoms and eight items assess anxiety symptoms. An example of a depressive symptom question is “Did you lose interest in most things?”. For each item, participants can answer “yes” if they have the symptom or “no” if they do not have the symptom. For both depression and anxiety a total score can be calculated by summing the items where the participants answer yes. For depression and anxiety the total score can range from zero to nine and from zero to eight, respectively.

#### Anxiety symptoms

The Generalized Anxiety Disorder-7 (GAD-7 [43]) is a valid and reliable seven-item measure assessing symptoms of generalized anxiety disorder. Respondents are asked to indicate the frequency of occurrence of a list of symptoms over the past 2 weeks on a four-point scale (1 = “not at all”, 2 = “several days”, 3 = “more than half the days”, and 4 = “nearly every day”). The overall score is the sum of the item scores and ranges from 7 to 28.

### Instrumental activities of daily living

Lawton’s Instrumental Activities of Daily Living Scale (IADL [44]) is a reliable instrument to assess everyday functional status. It describes seven activities including preparing meals, managing medications, and using the telephone. For each activity respondents have to rate how difficult they find performing the activity (0 = “no difficulty”, 1 = “some difficulty”, and 2 = “great difficulty”). The total score ranges from a possible 0 to 14.

### Perceived health

We assessed perceived health with a single-item question (taken from the SF-36 [45]) asking participants to rate their own health on a four-point scale ranging from excellent to poor (“excellent”, “good”, “fair”, and “poor”).

### Analyses

**Fig. 1:**
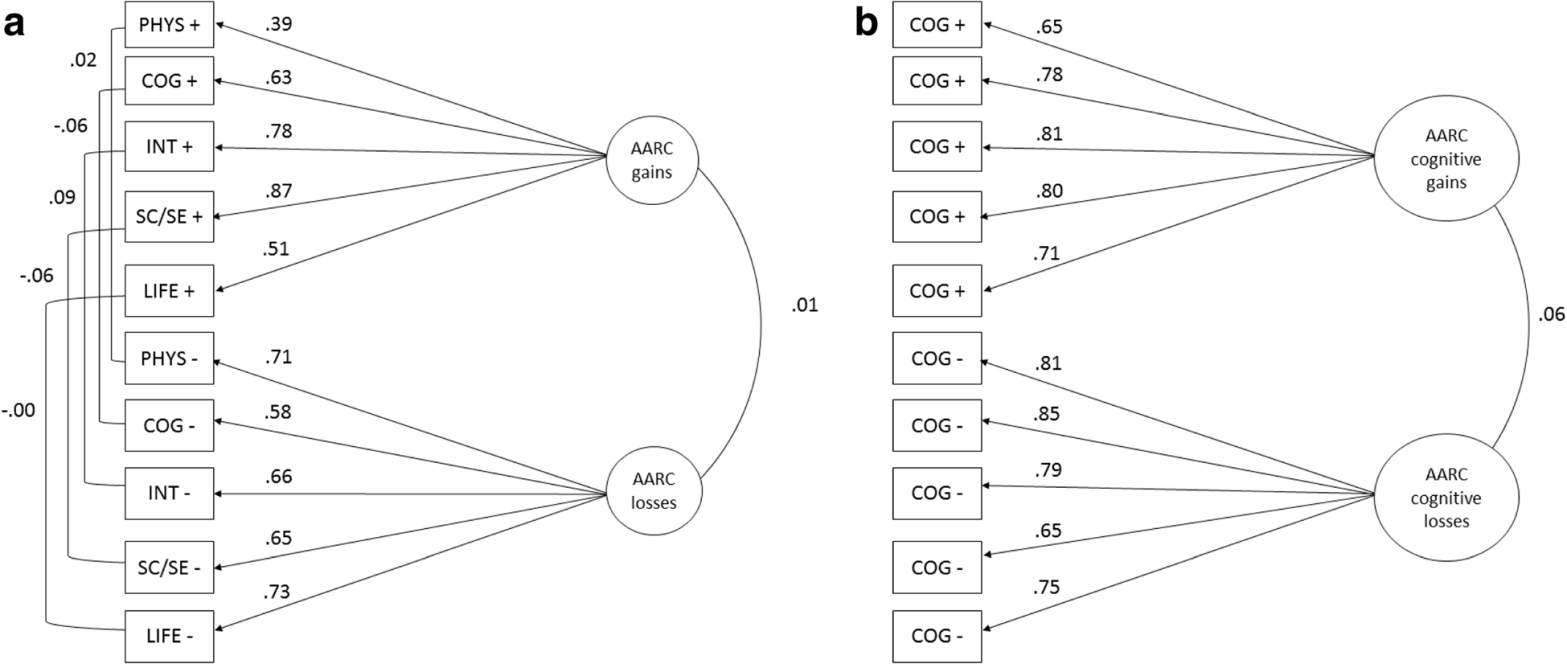
**a** Two-factor model of the AARC-10 SF. Measurement model of Awareness of Age-Related Changes (AARC) for the AARC-10 SF. Fully standardized coefficients are reported. AARC Domain abbreviations: PHY = Health and physical functioning; COG = Cognitive functioning; INT = Interpersonal relations; SCSE = Social-cognitive and social-emotional functioning; LIFE = Lifestyle and engagement; “+” = Positive domains; “-” = Negative domains. **b** Two-factor model of the AARC-50 cognitive functioning subscale. Measurement model of Awareness of Age-Related Changes (AARC) for the AARC-50 cognitive functioning subscale. Fully standardized coefficients are reported. COG = Cognition, “+” = Positive domains; “-” = Negative domains

As the validation of the AARC-10 SF [1] in US and German samples supported a two-factor structure (one factor for each of AARC gains and AARC losses), we used confirmatory factor analysis (CFA) to confirm this structure in the UK population. We tested whether the five items assessing gains and the five items assessing losses (of the AARC-10 SF) are related to the respective hypothesized underlying factors of AARC gains and AARC losses. The two factors AARC gains and AARC losses were allowed to correlate in the CFA model. Error terms were allowed to correlate for the pair of gains and losses items for the same AARC behavioral domain (Fig. 1).

CFA was also conducted to confirm the two-factor structure of the AARC-50 cognitive functioning subscale [8] (Fig. 1b). For both the AARC-10 SF and the AARC-50 cognitive functioning subscale, to confirm the need for a two-factor model (above described), we also fitted a model in which a single factor loaded on all ten items. For both the AARC-10 SF and the AARC-50 cognitive functioning subscale, we compared goodness of fit indices (GOF) of the two-factor model with those of one-factor model. Because the Chi-squared statistic is often significant for well-fitting models in large samples [46] alternative goodness of fit measures including the Comparative Fit index (CFI), the Tucker-Lewis index (TLI), the Root Mean Square Error of Approximation (RMSEA), and the Standardized Root Mean Square Residual (SRMR) were examined. Criteria for acceptable model fit were CFI and TLI > .90, RMSEA < .08 (90% CI: between 0 and .08), and SRMR < .06 [47]. The CFA models were fitted using the *sem* command in Stata. Analyses included only participants that provided complete data on all items.

We used Cronbach’s alpha (*α*) to quantify reliability for the gains and losses subscales of the AARC-10 SF and the AARC-50 cognitive functioning subscale [48]. We considered α values between .65 and .95 to be satisfactory.

For both the AARC-10 SF and the AARC-50 cognitive functioning subscale, we used CFA to test measurement invariance [18, 49, 50] between men and women, between two groups characterized by university education (vocational qualification, undegraduate degree, post-graduate degree, or doctorate) and no university education (secondary or post-secondary education, and among three age groups (middle age = 50 to 65 years; early old age = 66 to 75 years, advanced old age = 76 years and over). To explore measurement invariance, we fitted three CFA models: (a) Model 1 placed no equality constraints across groups on factor loadings, item intercepts, the error variances, the variances of the latent variables, or the covariances of the latent variables (assumes configural invariance); (b) Model 2 constrained the factor loadings to be identical across subgroups (assumes metric invariance); (c) Model 3 constrained the factor loadings and item intercepts to be identical across subgroups (assumes strong invariance).

To evaluate the fit of a model compared to a less restrictive one, the traditional approach involves assessing the differences in the Chi-squared fit statistics of the two examined CFA models by conducting likelihood ratio tests (LRT). However, as LRTs often result in statistically significant differences in large samples for models that are not markedly different in fit [46] and alternative fit indices are less sensitive to sample size [51], we explored model differences using alternative GOF indices including the Comparative Fit index (CFI), the Root Mean Square Error of Approximation (RMSEA), and the Standardised Root Mean Square Residual (SRMR). We concluded that a model had a worse fit than a less constrained model when the difference in CFI (ΔCFI) was larger than −.01 [52, 53], the difference in RMSEA (ΔRMSEA) was larger than .015 [54], and the difference in SRMR (ΔSRMR) was larger than .03 [54].

Construct validity for the AARC-10 SF was explored by estimating correlations between the AARC-10 SF and each of felt age, ATOA, measures of mental and physical health, and objective assessments of cognitive functioning (Grammatical Reasoning, Digit Span, Self-ordered Search, and Paired Associate Learning). Construct validity for the AARC-50 cognitive functioning subscale was explored by estimating correlations between the AARC-50 cognitive functioning subscale and each of felt age, ATOA, and objective (Grammatical Reasoning, Digit Span, Self-ordered Search, and Paired Associate Learning), self-reported, and informant-reported assessments of cognitive functioning. We used Pearson’s *r* to quantify correlations [55]. Correlation coefficients under .10 were considered negligible, between .10 to .29 were considered small, between .30 to .49 were considered moderate, and .50 or above were considered large [56].

To explore whether age, sex, marital status, employment status, and university education explain variability in levels of AARC gains and/or AARC losses, we fitted multiple linear regression models for each of the AARC-10 SF and the AARC-50 cognitive functioning gains and losses. We also conducted simple regressions in which the predictive role of each demographic variable (age, sex, marital status, employment status, and university education) on levels of AARC gains/losses was explored without controlling for the predictive role of the remaining demographic variables.

## Results

### Self-perceptions of aging among the study sample

On the AARC-10 SF the majority of participants reported moderate (20.7%), quite a bit of (47%), or a great deal of (27.8%) AARC gains and little (60.4%) or moderate (29%) AARC losses. On the AARC-50 cognitive functioning subscale the majority of participants reported moderate (39.6%) or quite a bit (28.0%) of AARC gains in their cognition and little (56.6%) or moderate (28.6%) AARC losses in their cognition. Further details about proportions of gains and losses perceived by participants on the AARC-10 SF and the AARC-50 cognitive functioning subscale are reported in Table 4. Seventeen percent of participants felt younger than their chronological age. Participants’ mean (SD) score on the ATOA scale was .52 (0.16) indicating that participants reported positive ATOA in some items but negative ATOA in others.

**Table 4:** Proportions of gains and losses reported by participants on the AARC-10 SF and the AARC-50 cognitive functioning subscale

	AARC-10 SF		AARC-50 cognitive functioning subscale	
	Gains	Losses	Gains	Losses
Not being aware of age-related changes	0.1%	3.8%	1.1%	5.7%
Little awareness of age-related changes	4.4%	60.4%	23.4%	56.6%
Moderate awareness of age-related changes	20.7%	29.0%	39.6%	28.6%
Quite a bit of awareness of age-related changes	47.0%	6.0%	28.0%	7.4%
A great deal of awareness of age-related changes	27.8%	0.8%	7.9%	1.9%

### Psychometric properties of the AARC-10 SF and AARC-50 cognitive functioning scale

#### Confirmatory factor analysis

For the AARC-10 SF, compared to a one-factor model (RMSEA = .21; 95% CI: .00, .00; CFI = .48; TLI = .33; SRMR = .18) the hypothesized two-factor model was a better fit as indicated by GOF indices (RMSEA = .07; 95% CI: .07, .07; CFI = .94; TLI = .92; SRMR = .05).

Item characteristics for the ten items of the AARC-10 SF are displayed in Table 5. The associations between factors and indicators were reasonably strong for all items (Fig. 1). Factor loadings for the individual domain items on the gains factor reflect greater heterogeneity of aging experiences in the gains compared to the losses factor.

**Table 5:** Item characteristics and Cronbach’s *α*s for the two subscales of the AARC-10 SF and the AARC-50 cognitive functioning subscale

	With my increasing age, I realize that…			
AARC-10 SF domain^a^		Basic item characteristics		Item-total correlation
		Mean	SD	
PHYS-	… I have less energy	2.8	1.1	.75
COG-	…my mental capacity is declining	2.1	.9	.77
INT-	…I feel more dependent on the help of others	1.5	.7	.76
SCSE-	…I find it harder to motivate myself	1.7	.8	.78
LIFE-	…I have to limit my activities	1.9	.9	.74
PHYS+	…I pay more attention to my health	3.1	1.1	.78
COG+	…I have more experience and knowledge to evaluate things and people	3.5	1.0	.71
INT+	…I appreciate relationship and people much more	3.8	1.1	.69
SCSE+	…I have a better sense of what is important for me	3.9	1.0	.67
LIFE+	…I have more freedom to live my days the way I want	3.7	1.2	.76
AARC-50 cognitive functioning		Basic item characteristics		Item-total correlation
		Mean	SD	
COG1 -	…my mental capacity is declining	2.1	.9	.84
COG2 -	...I am slower in my thinking	1.8	.8	.84
COG3 -	…I have a harder time concentrating	1.7	.8	.85
COG4 -	…learning new things takes more time and effort	2.4	1.0	.87
COG5 -	…I am more forgetful	2.2	1.0	.85
COG1 +	…I have more experience and knowledge to evaluate things and people	3.5	1.0	.86
COG2 +	…I have more foresight	2.7	1.1	.83
COG3 +	…I have become wiser	2.6	1.2	.82
COG4 +	…I think things through more carefully	2.6	1.1	.82
COG5 +	…I gather more information before I make decisions	2.6	1.1	.85

Note: ^a^AARC domain abbreviations: *PHY* Health and physical functioning, *COG* Cognitive functioning, *INT* Interpersonal relations, *SCSE* Social-cognitive and social-emotional functioning, *LIFE* Lifestyle and engagement; “+” = Positive domains; “-” = Negative domains

For the AARC-50 cognitive functioning subscale, compared to a one-factor model (RMSEA = .29; 95% CI = .00, .00; CFI = .42; TLI = .26; SRMR = .24) the hypothesized two-factor model was a better fit as indicated by GOF indices (RMSEA = .12; 95% CI: .12, .12; CFI = .90; TLI = .87; SRMR = .05). We calculated modification indices for the AARC-50 two-factor model; however modification indices did not suggest any pattern that would have significantly improved the model. Item characteristics for the 10 items of the AARC-50 cognitive functioning subscale are displayed in Table 5. The associations between construct and indicators were strong for all items (Fig. 1b).

#### Reliability

For the AARC-10 SF item-to-total score correlations had values between .67 and .78; hence all items reached satisfactory *α*s (Table 5). Cronbach’s *α* value was .77 for the AARC-10 SF gains scale and .80 for the AARC-10 SF losses scale. For the AARC-50 cognitive functioning subscale all item-to-total score correlations reached satisfactory values, ranging between .82 and .87 (Fig. 1b). Cronbach’s *α* value was .86 for the AARC-50 cognitive functioning subscale gains and .88 for the AARC-50 cognitive functioning subscale losses.

#### Measurement invariance between sex groups for the AARC-10 SF and the AARC-50 cognitive functioning subscale

With respect to measurement invariance for the AARC-10 SF between sex groups, compared to the model with all parameters freely estimated (assuming configural invariance), the model that restricted factor loadings to be the same across groups (assuming metric invariance) did not substantially reduce the GOF (Table 6). Hence, the meaning of the concepts of AARC-10 SF gains and AARC-10 SF losses appeared to be the same for men and women, allowing for valid representation of AARC gains and losses in correlational studies across men and women. Restricting item intercepts to be the same for men and women (assuming strong invariance) substantially decreased model fit as indicated by GOF indices, meaning that men and women interpret some items of the AARC-10 SF gains and the AARC-10 SF losses subscales differently; hence scores on single items cannot be compared among men and women. Since men and women systematically interpreted at least some items differently, responses for men and women should not be compared without taking this sex bias into account.

**Table 6:** Summary of the measurement invariance models for the AARC-10 SF and the AARC-50 cognitive functioning subscale-sex groups

AARC-10 SF			
Models	RMSEA [95% CI]	CFI	SRMR
Model 1: Configural invariance	.07 [.06, .07]	.95	.05
Model 2: Metric invariance	.06 [.06, .07]	.95	.05
Model 3: Strong invariance	.07 [.07, .08]	.92	.71
AARC-50 cognitive functioning subscale			
Models	RMSEA [95% CI]	CFI	SRMR
Model 1: Configural invariance	.12 [.12, .12]	.90	.05
Model 2: Metric invariance	.11 [.11, .12]	.90	.05
Model 3: Strong invariance	.11 [.11, .11]	.90	.10

CONFIGURAL INVARIANCE = This model places no equality constraints across groups on factor loadings, the error variances, the variances of the latent variables, or the covariances of the latent variables

METRIC INVARIACNE = This model places the factor loadings to be equal across groups

STRONG INVARIANCE = This model constrains the factor loadings and the item intercepts to be equal across groups

RMSEA = Root mean square error of approximation. CFI = Comparative fit index. SRMR = Standard root mean square residual

With respect to measurement invariance between sex groups for the AARC-50 cognitive functioning subscale, compared to the model with all parameters freely estimated in the men and women groups (assuming configural invariance), restricting factor loadings to be the same across groups (assuming metric invariance) did not substantially decrease model fit (Table 6). Hence, the meaning of the concept of AARC as captured by the AARC-50 cognitive functioning subscale appeared to be the same for men and women. Restricting item intercepts and factor loadings (assuming strong invariance) to be equal among men and women did not substantially decrease GOF indices (RMSEA, CFI, and SRMR) (Table 6), meaning that men and women interpret items of the AARC-50 cognitive functioning gains and losses subscales in the same way. Hence comparison of both observed total scores across items and estimated factor means between sex groups are possible.

#### Measurement invariance between groups defined by education level for the AARC-10 SF and the AARC-50 cognitive functioning subscale

With respect to measurement invariance for education level groups (university education versus no university education) for the AARC-10 SF, compared to the model with freely estimated parameters in the two groups (assuming configural invariance), restricting factor loadings to be the same across groups (assuming metric invariance) did not decrease model fit substantially (Table 7). Hence, the meaning of the concepts of AARC gains and AARC losses as captured by the AARC-10 SF appeared to be the same for people with and without a university education. Restricting item intercepts (assuming strong invariance) to be equal among groups with a university education and without a university education did not substantially decrease GOF indices (RMSEA, CFI, and SRMR) (Table 7), meaning that individuals with a university education interpret items of the AARC gains and AARC losses subscales as captured by the AARC-10 SF similarly to their counterparts without a university education. Hence for the AARC-10 SF comparison of both observed total scores across items and estimated factor means between education-based groups are possible.

**Table 7:** Summary of the measurement invariance models for the AARC-10 SF and the AARC-50 cognitive functioning subscale-education level

AARC-10 SF			
Models	RMSEA [95% CI]	CFI	SRMR
Model 1: Configural invariance	.07 [.07, .07]	.95	.05
Model 2: Metric invariance	.07 [.06, .07]	.95	.05
Model 3: Strong invariance	.06 [.06, .07]	.94	.06
AARC-50 cognitive functioning subscale			
Models	RMSEA [95% CI]	CFI	SRMR
Model 1: Configural invariance	.12 [.12, .12]	.91	.05
Model 2: Metric invariance	.11 [.11, .12]	.90	.99
Model 3: Strong invariance	.11 [.11, .11]	.90	.06

CONFIGURAL INVARIANCE = This model places no equality constraints across groups on factor loadings, the error variances, the variances of the latent variables, or the covariances of the latent variables

METRIC INVARIACNE = This model places the factor loadings to be equal across groups

STRONG INVARIANCE = This model constrains item loadings, error-variances of the items, variances

*RMSEA* Root mean square error of approximation, *CFI* Comparative fit index, *SRMR* Standard root mean square residual

With respect to measurement invariance for education level groups (university education versus no university education) for the AARC-50 cognitive functioning subscale, compared to the model that freely estimated parameters in the education groups (assuming configural invariance), the model restricting factor loadings to be the same (assuming metric invariance) across groups did not markedly decrease model fit (Table 7). Hence, the meaning of the concepts of AARC gains and AARC losses in the cognitive domain as captured by the AARC-50 cognitive functioning subscale appeared to be the same across people with a university education and without a university education. Restricting item intercepts (strong invariance) to be equal among participants with a university education and without a university education did not substantially decrease GOF indices (RMSEA, CFI, and SRMR) (Table 7), meaning that people with a university education and people without a university education interpret items of the AARC gains and AARC losses subscales captured by the AARC-50 cognitive functioning subscale in the same way. Hence for the AARC-50 cognitive functioning subscale comparison of both estimated factor means and observed total scores across items between education-based groups are possible.

#### Measurement invariance among groups in middle age, early old age, and advanced old age for the AARC-10 SF and the AARC-50 cognitive functioning subscale

With respect to measurement invariance among groups in middle age, early old age, and advanced old age (middle age = 50 to 65 years; early old age = 66 to 75 years; advanced old age = 76 years and over) for the AARC-10 SF, compared to the model with freely estimated parameters in the three age groups (assuming configural invariance), restricting factor loadings to be the same across groups (assuming metric invariance) did not decrease model fit substantially (Table 8). Hence, the meaning of the concepts of AARC gains and AARC losses as captured by the AARC-10 SF appeared to be the same across middle age, early old age, and advanced old age. Restricting item intercepts (assuming strong invariance) to be equal among age groups did not substantially decrease GOF indices (RMSEA, CFI, and SRMR) (Table 8), meaning that in middle age, early old age, and advanced old age individuals interpret items of the AARC gains and AARC losses subscales of the AARC-10 SF similarly. Hence for the AARC-10 SF comparisons of both observed total scores across items and estimated factor means among age groups are possible.

**Table 8:** Summary of the measurement invariance models for the AARC-10 SF and the AARC-50 cognitive functioning subscale-age groups

AARC-10 SF			
Models	RMSEA [95% CI]	CFI	SRMR
Model 1: Configural invariance	.07 [.07, .07]	.94	.07
Model 2: Metric invariance	.07 [.07, .07]	.94	.07
Model 3: Strong invariance	.08 [.08, .08]	.90	.10
AARC-50 cognitive functioning subscale			
Models	RMSEA [95% CI]	CFI	SRMR
Model 1: Configural invariance	.12 [.12, .12]	.91	.06
Model 2: Metric invariance	.11 [.11, .11]	.90	.06
Model 3: Strong invariance	.11 [.11, .11]	.89	.08

CONFIGURAL INVARIANCE = This model places no equality constraints across groups on factor loadings, the error variances, the variances of the latent variables, or the covariances of the latent variables

METRIC INVARIACNE = This model places the factor loadings to be equal across groups

STRONG INVARIANCE = This model constrains item loadings, error-variances of the items, variances

*RMSEA* Root mean square error of approximation, *CFI* Comparative fit index, *SRMR* Standard root mean square residual

With respect to measurement invariance among the three age groups for the AARC-50 cognitive functioning subscale, compared to the model that freely estimated parameters in the three age groups (assuming configural invariance), the model restricting factor loadings to be the same (assuming metric invariance) across the three age groups did not markedly decrease model fit (Table 8). Hence, the meaning of the concepts of AARC gains and AARC losses in the cognitive domain as captured by the AARC-50 cognitive functioning subscale appeared to be the same in middle age, early old age, and advanced old age. Restricting item intercepts (strong invariance) to be equal across the three age groups did not substantially decrease GOF indices (RMSEA, CFI, and SRMR) (Table 8), meaning that items of the AARC gains and AARC losses subscales of the AARC-50 cognitive functioning subscale are interpreted in the same way across middle age, early old age, and advanced old age. Hence for the AARC-50 cognitive functioning subscale comparisons of both estimated factor means and observed total scores across items among age groups are possible.

#### Validity of the AARC-10 SF and AARC-50 cognitive functioning scale in the over 50s UK population

Correlational evidence for validity of the AARC-10 SF is reported in Table 9. As expected, individuals who experience more AARC gains, assessed with the AARC-10 SF, feel younger (*r* = .10; 95% CI: .08 to .12) and have more positive ATOA (*r* = .12; 95% CI: .10 to .14) compared to individuals who experience fewer AARC gains. People who experience more AARC losses, assessed with the AARC-10 SF, feel older (*r* = −.27; 95% CI: −.29 to −.25) and have more negative ATOA (*r* = −.23; 95% CI: −.25 to −.21) compared to individuals who experience fewer AARC losses. Overall, we found mixed and negligible correlations between AARC gains, assessed with the AARC-10 SF, and indicators of mental and physical health. Individuals who experience higher AARC losses, assessed with the AARC-10 SF, score higher on measures assessing current symptoms of depression (*r* = .21; 95% CI: .19 to .23) and anxiety (*r* = .32; 95% CI: .30 to .34), as well as lifetime symptoms of depression (*r* = .13; 95% CI: .12 to .16) and anxiety (*r* = .16; 95% CI: .14 to .18).

**Table 9:** Correlations between AARC-10 SF and measures of self-perceptions of aging, mental and physical health

Correlational evidence of validity of the AARC-10 SF						
	AARC-10 SF Gains			AARC-10 SF Losses		
	Pearson’s *r*	[95% CI]	*p*-value	Pearson’s *r*	[95% CI]	*p*-value
Felt age	.10	[.08, .12]	< .001	−.27	[−.29, −.25]	< .001
CIDI-Lifetime depressive symptoms	.07	[.05, .09]	< .001	.13	[.12, .16]	< .001
CIDI-Lifetime anxiety symptoms	.04	[.02, .06]	< .001	.16	[.14, .18]	< .001
GAD-7	−.03	[−.05, −.01]	.01	.21	[.19, .23]	.01
PHQ-9	−.08	[−.10, −.06]	< .001	.32	[.30, .34]	< .001
IADL	−.03	[−.05, −.01]	< .001	.23	[.21, .24]	< .001
Perceived health	.09	[.08, .12]	< .001	−.44	[−.46, −.43]	< .001
Digit span	−.01	[−.04, .01]	.09	−.12	[−.14, −.09]	< .0001
Paired associate learning	−.01	[−.04, .01]	.32	−.11	[−.14, −.09]	< .0001
Verbal reasoning	−.04	[−.07, −.02]	.001	−.15	[−.18, −.13]	< .0001
Self-ordered search	−.05	[−.08, −.03]	< .0001	−.10	[−.12, −.08]	< .0001
	Spearman’s *ρ*		*p*-value	Spearman’s *ρ*		*p*-value
ATOA	.13		< .0001	−.25		< .0001

*AARC-10 SF gains* Subscale of the AARC-10 SF assessing AARC gains, *AARC-10 SF losses* Subscale of the AARC-10 SF assessing AARC losses, *Felt age* Felt age discrepancy score between participants’ chronological age and the age they feel they are, *ATOA* Lawton’s attitudes toward own aging 5-item scale, *CIDI-Lifetime depressive symptoms* Composite international diagnostic interview-depressive symptoms, *CIDI-Lifetime anxiety symptoms* Composite international diagnostic interview-anxiety symptoms. *GAD-7* Generalized anxiety disorder-7, *PHQ-9* Patient Health Questionnaire-9, *IADL* Lawton’s Instrumental activities of daily living scale. Perceived health = Participants rated their own health on a four-point scale ranging from excellent to poor “excellent”, “good”, “fair”, and “poor”

Participants with better functioning in activities of daily living and who rate their health more positively experience higher levels of AARC gains, assessed with the AARC-10 SF, but these correlations are negligible. Participants with better functioning in activities of daily living (*r* = .23; 95% CI: .21 to .24) and who rate their health more positively (*r* = −.44; 95% CI: −.46 to −.43) experience lower levels of AARC losses, assessed with the AARC-10 SF, than participants with worse functional health and who rate their health more negatively.

Correlations of the cognitive tasks digit span (*r* = −.01; 95% CI: −.04 to .01) and paired associate learning (*r* = −.01; 95% CI: −.04 to .01) with AARC gains assessed with the AARC-10 SF were not significant. The cognitive tasks verbal reasoning (*r* = −.04; 95% CI: −.07 to −.02) and self-ordered search (*r* = −.05; 95% CI: −.08 to −.03) showed negative and negligible associations with AARC gains assessed with the AARC-10 SF. The cognitive tasks digit span (*r* = −.12; 95% CI: −.14 to −.09), paired associate learning (*r* = −.11; 95% CI: −.14 to −.09), verbal reasoning (*r* = −.15; 95% CI: −.18 to −.13), and self-ordered search (*r* = −.10; 95% CI: −.12 to −.08) showed negative small associations with AARC losses assessed with the AARC-10 SF.

#### Validity of the AARC-10 SF and AARC-50 cognitive functioning scale in the over 50s UK population

Correlational evidence for validity of the AARC-50 cognitive functioning subscale is reported in Table 10. We found that those individuals who have higher awareness of negative changes in their cognitive functioning also feel older and have more negative ATOA than those individuals having lower AARC losses. Regarding the correlations between the AARC-50 cognitive functioning subscale and objective cognitive tasks, when compared to individuals with fewer AARC gains, individuals with higher levels of awareness of positive changes score worse in tasks assessing digit span, verbal reasoning, and self-ordered search; estimate that their cognitive abilities have increased over the past ten years; and a person close to them also estimates that their abilities have increased over the past ten years. However, most of the above described correlations were of negligible size. Compared to individuals with higher scores on cognitive tests, individuals with lower scores on cognitive tests experience higher levels of negative age-related changes; correlations among AARC losses and cognitive tasks were, however, either negligible or small. Participants who report higher levels of negative age-related changes in cognition also notice a decrease in their cognitive abilities over the past ten years and this correlation was the strongest in size (*r* = .47; 95% CI: .45 to .49). However, participants’ awareness of negative age-related changes is not associated with the judgment of an informant.

**Table 10:** Correlations between AARC-50 cognitive functioning subscale and measures of self-perceptions of aging, subjective and objective cognition

Correlational evidence of validity of the AARC-50 cognitive functioning subscale						
	AARC-50 cognitive functioning gains			AARC-50 cognitive functioning losses		
	Pearson’s *r*	[95% CI]	*p*-value	Pearson’s *r*	[95% CI]	*p*-value
Felt age	.08	[.06, .10]	< .001	−.19	[−.21, −.17]	< .001
Digit span	−.05	[−.08, −.03]	< .001	−.10	[−.12, −.07]	< .001
Paired associate learning	−.02	[−.05, .00]	.06	−.11	[−.14, −.09]	< .001
Verbal reasoning	−.09	[−.12, −.07]	< .001	−.16	[−.18, −.13]	< .001
Self-ordered search	−.07	[−.10, −.05]	< .001	−.08	[−.11, −.06]	< .001
IQCODE informant	−.05	[−.07, −.03]	< .001	−.01	[−.01, .03]	.51
IQCODE self	−.12	[−.15, −.10]	< .001	.47	[.45, .49]	< .001
	Spearman’s *ρ*		*p*-value	Spearman’s *ρ*		*p*-value
ATOA	.04		< .0001	−.14		< .0001

AARC-50 cognitive functioning gains = Subscale of the AARC 50-item questionnaire assessing gains in the cognitive functioning domain. AARC-50 cognitive functioning losses = Subscale of the AARC 50-item questionnaire assessing losses in the cognitive functioning domains. Felt age = Felt age discrepancy score between participants’ chronological age and the age they feel they are. ATOA = Lawton’s attitudes toward own aging 5-item scale. Digit span = Computerized cognitive task assessing verbal working memory. Paired associate learning = Computerized cognitive task assessing visual episodic memory. Grammatical reasoning task = Computerized cognitive task assessing verbal reasoning. Self-oriented search = Computerized cognitive task assessing spatial working memory. IQCODE informant = Informant Questionnaire on Cognitive Decline in the Elderly short form asking informants to rate the cognitive change of someone close to the them over the last 10 years. IQCODE self = Informant Questionnaire on Cognitive Decline in the Elderly short form asking participants to rate their own cognitive change over the last 10 years

As the correlation between participants’ perceptions of negative age-related changes in cognition and perceptions of a decrease in cognition over the past ten years was the only correlation of moderate size, we further explored whether the size of the correlation varies across different age-groups (middle age = 50 to 65 years; early old age = 66 to 75 years, advanced old age = 76 years and over). We found that the size of the correlation is similar among participants in middle age (*r* = .48; 95% CI: .45 to .51); early old age (*r =* .47; 95% CI: .44 to .50); and advanced old age (*r* = .42; 95% CI: .34 to .91).

#### Demographic variables as predictors of the AARC-10 SF and AARC-50 cognitive functioning subscale

From the two multiple regressions exploring the ability of demographic variables to predict gains and losses measured on the AARC-10 SF (Tables 11 and 12), we found that, overall, being older, employed, and having a university education significantly predict lower levels of AARC gains; while being a woman significantly predicts higher levels of AARC gains. We also found that being a woman, married, in a civil partnership, or co-habiting, and having a university education significantly predict fewer AARC losses; while being older significantly predicts more AARC losses.

**Table 11:** Simple and multiple regressions with demographic variables as predictors of AARC gains scores on the AARC-10 SF

(*N* = 8639)	Demographic variables as predictors of AARC gains: Simple regressions				Demographic variables as predictors of AARC gains: Multiple regression			
	AARC-10 SF losses							
Variables	Coeff.	[95% CI]	*p*-value	Standardized Coeff.	Coeff.	[95% CI]	*p*-value	Standardized Coeff.
Age	−.02	[−.04, −.01]	< .0001	−.05	−.03	[−.04, −.01]	< .001	−.05
Sex	1.40	[1.21, 1.60]	< .0001	.15	1.31	[1.11, 1.51]	< .001	.14
Marital status	−.39	[−.59, −.19]	< .0001	−.04	−.27	[−.48, −.06]	.01	−.03
Employment	.04	[−.12, .21]	.60	.01	−.23	[−.43, −.03]	.02	−.03
University education	−.25	[−.44, −.06]	.01	−.03	−.22	[−.41, −.03]	.02	−.02
Total *R*^*2*^					.03			
Adjusted *R*^*2*^					.02			
*Model F-test*					44.61 (5, 8633); *p* < .001			

Note: In the regression models we included only those participants that have no missing data. AARC-10 SF gains = Subscale of the AARC-10 SF assessing AARC gains. Marital Status was operationalized as a dichotomous variable capturing whether the participant is married/ civil partnership/ co-habiting or widowed/ separated/ divorced/ single. Employment was operationalized as a dichotomous variable capturing whether the participant is working or not. University education was operationalized as a dichotomous variable. Standardized beta coefficients are calculated by subtracting the mean from the variable and dividing it by its standard deviation

**Table 12:** Simple and multiple regressions with demographic variables as predictors of AARC losses scores on the AARC-10 SF

(*N* = 8639)	Demographic variables as predictors of AARC losses: Simple regressions				Demographic variables as predictors of AARC losses: Multiple regression			
	AARC-10 SF losses							
Variables	Coeff.	[95% CI]	*p*-value	Standardized Coeff.	Coeff.	[95% CI]	*p*-value	Standardized Coeff.
Age	.11	[.10, .12]	< .0001	.24	.09	[.08, .11]	< .001	.20
Sex	−.80	[−.96, −.64]	< .0001	−.10	−.60	[−.76, −.44]	< .001	−.08
Marital status	−.78	[−.95, −.61]	< .0001	−.10	−.52	[−.68, −.35]	< .001	−.06
Employment	−.96	[−1.1, −.82]	< .0001	−.15	−.11	[−.28, .05]	.18	−.02
University education	−.45	[−.60, −.29]	< .0001	−.06	−.35	[−.50, −.19]	< .001	−.05
Total *R*^*2*^					.07			
Adjusted *R*^*2*^					.07			
*Model F-test*					128.74 (5, 8633); *p* < .001			

Note: In the regression models we included only those participants that have no missing data. AARC-10 SF losses = Subscale of the AARC-10 SF assessing AARC losses. Marital Status was operationalized as a dichotomous variable capturing whether the participant is married/ civil partnership/ co-habiting or widowed/ separated/ divorced/ single. Employment was operationalized as a dichotomous variable capturing whether the participant is working or not. University education was operationalized as a dichotomous variable. Standardized beta coefficients are calculated by subtracting the mean from the variable and dividing it by its standard deviation

From the multiple regressions exploring the ability of demographic variables to predict gains and losses measured on the AARC-50 cognitive functioning subscale (Tables 13 and 14), we found that, overall, being older, married, in a civil partnership, or co-habiting, and having a university education significantly predict fewer AARC gains; while being a woman and employed significantly predict more AARC gains. We also found that being a woman, employed, and having a university education significantly predict fewer AARC losses; while being older significantly predicts more AARC losses.

**Table 13:** Simple and multiple regressions with demographic variables as predictors of gains scores on the AARC-50 cognitive functioning subscale

(N = 8639)	Demographic variables as predictors of AARC gains: Simple regressions				Demographic variables as predictors of AARC gains: Multiple regression			
	AARC-50 cognitive functioning gains							
Variables	Coeff.	[95% CI]	*p*-value	Standardized Coeff.	Coeff.	[95% CI]	*p*-value	Standardized Coeff.
Age	−.06	[−.07, −.05]	< .0001	−.10	−.04	[−.06, −.03]	< .001	−.07
Sex	1.30	[1.08, 1.52]	< .0001	.12	1.06	[.84, 1.29]	< .001	.10
Marital status	−.51	[−.74, −.28]	< .0001	−.05	−.51	[−.75, −.28]	< .001	−.05
Employment	.78	[.59, .96]	< .0001	.09	.41	[.18, .63]	< .001	.05
University education	−.63	[−.84, −.41]	< .0001	−.06	−.63	[−.85, −.42]	< .001	−.06
Total *R*^*2*^					.03			
Adjusted *R*^*2*^					.03			
*Model F-test*					51.36 (5, 8633); *p* < .001			

Note: In the regression models we included only those participants that have no missing data. AARC-50 cognitive functioning gains = Subscale of the AARC 50-item questionnaire assessing gains in the cognitive functioning domain. Marital status was operationalized as a dichotomous variable capturing whether the participant is married/ civil partnership/ co-habiting or widowed/ separated/ divorced/ single. Employment was operationalized as a dichotomous variable capturing whether the participant is working or not. University education was operationalized as a dichotomous variable. Standardized beta coefficients are calculated by subtracting the mean from the variable and dividing it by its standard deviation

**Table 14:** Simple and multiple regressions with demographic variables as predictors of losses scores on the AARC-50 cognitive functioning subscale

(N = 8639)	Demographic variables as predictors of AARC losses: Simple regressions				Demographic variables as predictors of AARC losses: Multiple regressions			
	AARC-50 cognitive functioning losses							
Variables	Coeff.	[95% CI]	*p*-value	Standardized Coeff.	Coeff.	[95% CI]	*p*-value	Standardized Coeff.
Age	.08	[.07, .09]	< .001	.17	.07	[.05, .08]	< .001	.13
Sex	−.88	[−1.06, −.70]	< .001	−.11	−.76	[−.95, −.58]	< .001	−.09
Marital status	−.33	[−.51, −.14]	< .001	−.04	−.17	[−.36, .03]	.09	−.02
Employment	−.84	[−.99, −.69]	< .001	−.12	−.24	[−.43, −.06]	.01	−.03
University education	−.47	[−.64, −.30]	< .001	−.06	−.41	[−.59, −.24]	< .001	−.05
Total *R*^*2*^					.04			
Adjusted *R*^*2*^					.04			
*Model F-test*					70.07 (5, 8633); *p* < .001			

Note: In the regression models we included only those participants that have no missing data. AARC-50 cognitive functioning losses = Subscale of the AARC 50-item questionnaire assessing losses in the cognitive functioning domains. Marital status was operationalized as a dichotomous variable capturing whether the participant is married/ civil partnership/ co-habiting or widowed/ separated/ divorced/ single. Employment was operationalized as a dichotomous variable capturing whether the participant is working or not. University education was operationalized as a dichotomous variable.. Standardized beta coefficients are calculated by subtracting the mean from the variable and dividing it by its standard deviation

Tables 12 , 13 and 14 also show the results of simple regressions with each demographic variable (age, sex, marital status, current employment status, and university education) as a predictor of AARC gains and losses measured with the AARC 10-SF and the AARC-50 cognitive functioning subscale.

## Discussion

This was the first study exploring psychometric properties of the AARC-10 SF and the AARC-50 cognitive functioning subscale in the UK population. We found that both scales are valid and reliable measures of AARC gains and AARC losses in the UK population aged 50 and over, that can be used in correlational studies and in studies comparing AARC across men and women, across individuals with and without a university degree, and across middle age, early old age, and advanced old age. However, some caution should be exercised when comparing the scores of men and women on the AARC-10 SF. Both scales showed good convergent validity for AARC losses, but weak convergent validity for AARC gains. Finally, we found that age, sex, marital status, employment, and university education explained significant variability in levels of perceived AARC gains and losses assessed with the AARC-10 SF and the AARC-50 cognitive functioning subscale.

Factor loadings for the two-factor model of the AARC-10 SF and for the two-factor model of the AARC-50 cognitive functioning subscale were similar to those found in the US and German validations of the measures [1, 8], further supporting the use of these AARC measures in the UK. Also in line with previous validations of AARC measures, we found small and moderate overlap between AARC questionnaires (AARC-10 SF and AARC-50 cognitive functioning subscale) and measures assessing the way in which individuals experience aging (felt age and ATOA), supporting the conceptual distinction of AARC from similar concepts [1, 8]. The partial overlap of AARC (assessed with both the AARC-10 SF and AARC-50 cognitive functioning subscale) with felt age and ATOA suggests that AARC may impact on the way in which individuals feel older or younger than they are or how changes are reported or appreciated [57, 58]. However, ATOA and felt age may also be associated with individuals’ perceptions of AARC, such as perceptions of age-related cognitive changes. A recent longitudinal study showed that more negative ATOA predict greater perceived cognitive decline [59]. Similarly, individuals with an older felt age perceive more age-related losses in the cognitive domain [60]. Overall, results relating to the construct validity of the AARC-10 SF and the AARC-50 cognitive functioning subscale suggest that levels of AARC losses and AARC gains are informative of individuals’ mental, physical, and cognitive health. The correlation we found between higher levels of AARC-10 SF losses and more symptoms of depression is in line with previous evidence describing the predictive role of higher perceived AARC losses over higher levels of depressive symptoms (e.g., [1, 8, 9]). Many of the variables contributing to the aetiology of late-life onset of depression, such as poor physical health [61], increased dependence on others [62], and having little or no social support, are normative aspects of older age. This may explain why the experiences of age-related losses that are captured in individuals’ perceptions of AARC losses are associated with current and future levels of depressive symptoms [63, 64].

This was the first study exploring correlations between AARC gains and losses and anxiety. Our results have shown that higher levels of AARC-10 SF losses are correlated with anxiety. Symptoms of anxiety may be expected in older age and may be a consequence of the negative changes that people experience in older age [26]. In contrast, the correlations we found between AARC-10 SF gains and symptoms of depression and anxiety were mixed and negligible. As similar findings were reported in previous studies [63–65], along with the newly-identified correlation with anxiety, it may be that when promoting mental health in older age decreasing AARC losses is more important than increasing AARC gains.

The positive correlation we found between IADL and AARC losses is in line with previous studies showing that individuals with poorer everyday functioning perceive more AARC losses [1]. Hence, individuals’ perceptions of AARC losses accurately reflect the negative changes that individuals experience in their lives. The finding that individuals with higher AARC gains and/or lower AARC losses (assessed with the AARC-10 SF) rate their health more positively is also in line with previous evidence [1, 8]. Most correlations between AARC (assessed with the AARC-10 SF) and indicators of health were small or moderate, suggesting the presence of multiple factors alongside AARC gains and AARC losses that may contribute to experiences of aging and levels of mental and physical health. The size of the correlations of AARC (assessed with the AARC-10 SF) with indicators of mental and physical health were stronger for AARC losses than for AARC gains and this is in line with existing literature on AARC [2, 65]. It may be that mental and physical health exert a greater influence on individuals’ perceptions of AARC losses than on the perceptions of AARC gains [2]. Perceptions of AARC gains may instead be more influenced by other factors such as personality traits [66], expectations for the future [12], and perceived social support [67].

This was the first study exploring correlations of AARC in the cognitive functioning domain (assessed with the AARC-50 cognitive functioning subscale) with objective, subjective, and informant-rated measures of cognition. We found that higher levels of AARC losses in the cognitive domain reflect lower objective cognitive performance and more negative self-evaluations of cognitive changes over 10 years. Moreover, the correlation between AARC losses in the cognitive domain and perceived cognitive change over the past ten years remained consistent across three age groups (aged 50 to 65; aged 66 to 75; and aged 76 and over), suggesting that the AARC-50 cognitive functioning subscale may detect across middle age, early old age, and advanced old age subclinical cognitive decline that is incorporated into individuals’ ratings of their AARC [1]. This finding is in line with evidence supporting the value of subjective cognitive complaints in informing about objective cognitive decline [10, 68]. We also found that participants’ experience of negative age-related changes is not correlated with informants’ rating of participants’ change in cognitive abilities over ten years. It may therefore be that cognitively healthy individuals are aware of the subtle cognitive changes they are experiencing but that such changes are unnoticed by people close to them [69].

Interestingly, those individuals who performed more poorly on objective cognitive tasks not only reported higher levels of AARC losses but also reported higher levels of AARC gains (assessed with both the AARC-10 SF and AARC-50 cognitive functioning subscale). It may be that in order to compensate for negative changes in cognition individuals engage in new cognitively stimulating activities [70, 71], resulting in increased self-perception of gains. Alternatively, reporting high levels of gains alongside high levels of losses may be a strategy of emotional coping: high levels of losses may cause mental distress which can be compensated for by directing thoughts towards positive age-related change [70]. However, the strength of the correlations of AARC gains with objective cognitive tasks and self-perceived cognitive decline were either of negligible size or small; self-perceptions of age-related gains in cognition may be influenced by individuals’ beliefs about aging, more than by individuals’ actual cognitive functioning. Overall, as most of the associations of AARC gains with cognitive indicators are either small or of negligible size, evidence for convergent validity for the AARC gains assessed with the AARC-50 cognitive functioning subscale is weaker than evidence for AARC losses.

A secondary aim of this study was to explore whether demographic variables (age, sex, marital status, current employment status, and university education) predict scores on the AARC-10 SF and AARC-50 cognitive subscale gains and losses. We found that the demographic variables age, sex, marital status, employment, and university education explain some variability in levels of AARC. We found that being older predicts fewer AARC gains and more AARC losses both in the AARC-10 SF and in the AARC-50 cognitive functioning subscale; the association of higher AARC losses with being older is in line with previous evidence and with gerontological literature reporting the greater salience of perceived losses among older individuals [12, 72]. The association between being older and fewer AARC gains is not consistent with previous evidence reporting a positive association between older age and higher levels of AARC gains [12]; this discrepancy in results may be due to cultural differences as the present study included UK residents whereas Brothers, Gabrian [12] included US and German participants. The associations of older age with lower AARC gains but higher AARC losses may be due to older individuals having a poorer health status than younger individuals [73, 74].

We also found that being a man predicts fewer AARC gains and more AARC losses both in the AARC-10 SF and in the AARC-50 cognitive functioning subscale. This finding is also in line with existing evidence on sex differences in AARC and in subjective well-being, showing that men report fewer AARC gains, higher AARC losses, and lower levels of subjective well-being [17, 25, 75] than women. This may be due to men being less actively focused on positive changes compared to women [76, 77]. Indeed, research shows that positive experiences of aging among women outweigh negative experiences, despite women being aware of significant changes in their body due to menopause [78].

In line with existing literature [75, 79, 80], we found that being married, in a civil relationship, or co-habiting predicts fewer AARC losses assessed both with the AARC-10 SF and with the AARC-50 cognitive functioning subscale. However we also found that being married, in a civil partnership, or co-habiting predicts lower levels of awareness of positive age-related change assessed both with the AARC-10 SF and with the AARC-50 cognitive functioning subscale. Literature on the role of marriage in relation to cognitive abilities is heterogeneous with some studies reporting lower cognitive abilities among non-married individuals [81] and conversely, others report a non-significant association between marital status and cognition [82].

Our results suggest that working may have distinct effects on different AARC life domains. Working predicted fewer AARC losses in cognition (as assessed with the AARC-50 cognitive functioning subscale) compared to non-working and this may be due to work stimulating cognition. Conversely, working predicted fewer AARC gains in the remaining AARC life domains (assessed with the AARC-10 SF) and this may be due to non-working individuals having more leisure time to enjoy hobbies and friends compared to workers, resulting in increased likelihood of experiencing age-related gains.

We found that people with a university education experience fewer AARC losses, but at the same time also experience fewer AARC gains assessed both with the AARC-10 SF and the AARC-50 cognitive functioning subscale compared to individuals without a university education. The fewer AARC losses in the cognitive domain experienced by those with a university education may be due to such individuals experiencing lower objective cognitive decline [83]. Indeed education exerts a protective role against cognitive decline [84, 85]. The lower score on the AARC-10 SF losses among those with a university education may be due to more highly educated people being more likely to engage in healthy behaviors and therefore to enjoy better physical health [86–89] and longer life expectancy [90].

An explanation for the lower levels of both AARC gains and losses on the AARC-10 SF and on the AARC-50 cognitive functioning subscale reported by those with a university education may be that individuals who experience low levels of age-related losses are less likely to reflect on age-related changes and as a consequence are less aware of positive age-related changes. However, we found that for both the AARC-10 SF and the AARC-50 cognitive functioning subscale correlations between AARC gains and AARC losses are negligible, indicating that there is no overall AARC. The lower levels of AARC gains reported by individuals with a university education may be due to more educated individuals attributing positive changes to other causes rather than to their increased age.

The study has limitations that need to be acknowledged. The sample included mainly white participants, women, individuals who were married (or in a civil partnership or co-habiting) and who had above average education and self-reported health. Among the 14,797 participants that took part in the PROTECT annual assessment between 1st January 2019 and 31st March 2019, 9410 participants completed the AARC questionnaires. Compared to those who did not complete the AARC questionnaires in 2019, the study sample included a larger proportion of women and participants who were better educated and a lower proportion of individuals who were employed. This selection bias may impact on study results such as on the predictive role that we found for being female and having a university level education over fewer AARC losses. However, there is no immediate reason to believe that the relationship between the predictors and AARC losses is different between those who provided data and those who did not.

Data for objective cognitive assessments were not collected on the same day on which participants completed the AARC questionnaires, but were completed within 2 months of AARC completion. This was because completing a battery of cognitive tasks is demanding, especially for older individuals, hence allowing participants to complete objective cognitive assessments on a separate day from the remaining measures decreased participants’ burden and increased the likelihood of collecting accurate answers. Moreover, cognitive functions do not deteriorate or deteriorate minimally in individuals without dementia over 2 months (e.g., [91, 92]). While cognitive abilities were assessed both through objective and subjective measures, mental and physical health were assessed through self-report measures only. Finally, individuals who completed a vocational qualification (e.g. diploma or certificate) were considered to have the same level of education as participants who completed a undergraduate degree, a master’s degree, or a doctorate. This is a limitation as several types of vocational qualifications exist, with some vocational qualifications being comparable to a university level education while others are not. However, it was not possible to classify participants’ education in a more detailed manner as PROTECT participants were not asked to specify the type of vocational qualification they obtained.

Despite the above limitations this study has a large sample size including a wide age range of UK participants. This is the first study testing content validity of the AARC-50 cognitive functioning subscale with subjective and objective measures of cognitive health. The quantification of psychometric properties based on a sample of participants without a diagnosis of dementia is important because in this study AARC losses in cognition (assessed with the AARC-50 cognitive functioning subscale) are associated with objective measures of cognition and therefore could be useful to identify early cognitive decline, which could in turn support efforts to prevent dementia. The AARC-50 cognitive functioning subscale could therefore potentially be used to identify those segments of the population at greater risk of cognitive decline who require closer cognitive monitoring, and may benefit from early intervention such as cognitive training programs [1] (pg. 3) or interventions that help them to accept their age-related changes and to minimize the negative impact of age-related cognitive changes [93]. The validation of the AARC-50 cognitive functioning subscale also makes it possible to conduct future research to better understand the cross-sectional relationship of subjective perceptions of cognition with objective cognitive functioning, as well as the longitudinal association with objective cognitive decline. Whereas much research studying perceived cognitive decline as a predictor of objective cognitive decline exists [10, 94, 95], the AARC-50 cognitive functioning subscale is particularly useful as it makes it possible to explore for the first time whether AARC gains convey protection against cognitive decline.

This is also the first study exploring content validity for the AARC-10 SF with a measure assessing perceived symptoms of anxiety, in addition to symptoms of depression, perceived health, and functional abilities that have been explored in the US and German validations of the AARC-10 SF and the AARC-50 cognitive functioning subscale [1, 8]. Good psychometric properties of the AARC-10 SF make it possible to use this scale to assess positive and negative perceptions of age-related changes in several domains of one’s life in clinical and research contexts within the UK.

## Conclusion

The AARC-10 SF is a valid and reliable measure to identify segments of the population that experience substantial change across multiple life domains as a consequence of their aging process. The brief measure may also be useful in clinical and counselling settings within UK to identify those individuals who, because of higher levels of AARC losses and/or lower levels of AARC gains, may benefit from interventions helping them to understand their age-related changes, to adapt to age-related changes, or to engage in healthy behaviors counteracting age-related losses [1].

The AARC-50 cognitive functioning subscale – while capturing a more narrow facet of the experience of aging - also proved to be a valid and reliable measure that could be used to identify those segments of the population at greater risk of cognitive decline and that may require closer cognitive monitoring or may benefit from early intervention such as cognitive training programs [1, 96]. Finally, as we found that demographic variables play a role in the experience of AARC gains and AARC losses, future studies on AARC should give a more detailed account of the mechanisms that foster the experience of age-reated gains or losses.

## Supplementary information

**Additional file 1 Supplementary Table 1** Mean and standard deviation on the four objective cognitive tasks for five levels of awareness of negative age-related cognitive changes

## Data Availability

This study was conducted using secondary data collected as part of the PROTECT ongoing study. PROTECT data are available to investigators outside the PROTECT team after request and approval by the PROTECT Steering Committee. Data for the AARC-10 SF questionnaire and for the AARC-50 cognitive functioning subscale will be available from May 2022.

## Abbreviations

AARC: Awareness of age-related changes
AARC gains: Awareness of positive age-related changes
AARC losses: Awareness of negative age-related changes
AARC-10 SF: 10-item questionnaire assessing awareness of age-related changes
AARC-50: 50-item questionnaire assessing awareness of age-related changes
PROTECT: Platform for research online to investigate genetics and cognition in aging (30)
ATOA: Attitudes Toward Own Aging
IQCODE: The Informant Questionnaire on Cognitive Decline in the Elderly short form
IQCODE- Self: The Informant Questionnaire on Cognitive Decline in the Elderly short form, self-version
PHQ-9: The Patient Health Questionnaire-9
CIDI-SF: The Composite International Diagnostic Interview-Short Form
GAD-7: The Generalized Anxiety Disorder-7
IADL: Lawton’s Instrumental Activities of Daily Living Scale
CFA: Confirmatory factor analysis
CFI: Comparative fit index
TLI: Tucker-Lewis index
RMSEA: Root mean square error of approximation
SRMR: Standardised root mean square residual
GOF: Goodness of fit
LRT: Likelihood ratio tests
ΔRMSEA: Difference in root mean square error of approximation
ΔSRMR: Difference in standardised root mean square residual
M: Mean
SD: Standard deviation
