# Supplementary material for: International relevance of two measures of awareness of age-related change (AARC)
Source: BMC Geriatr. 2020 Sep 21;20:359. doi: 10.1186/s12877-020-01767-6 (PMC7507664; doi:10.1186/s12877-020-01767-6)
Supplement: Supplementary file 1 — Additional file 1 Supplementary Table 1 Mean and standard deviation on the four objective cognitive tasks for five levels of awareness of negative age-related cognitive changes [file 12877_2020_1767_MOESM1_ESM.docx]

**Supplementary material**

**Supplementary Table 1: Mean and standard deviation on the four objective cognitive tasks for five levels of awareness of negative age-related cognitive changes**

| Level of negative age-related cognitive changes | N | Digit span; mean (SD) | Paired associate learning; mean (SD) | Verbal reasoning; mean (SD) | Self-ordered search; mean (SD) |
| --- | --- | --- | --- | --- | --- |
| Not at all aware of negative cognitive changes | 535 | 7.9 (1.9) | 4.9 (1.0) | 39.7 (10.3) | 7.9 (2.7) |
| A little aware of negative cognitive changes | 5331 | 7.7 (1.5) | 4.8 (0.9) | 38.4 (10.2) | 7.8 (2.6) |
| Moderately aware of negative cognitive changes | 2695 | 7.5 (1.5) | 4.6 (0.9) | 36.2 (10.8) | 7.5 (2.7) |
| Quite a bit aware of negative cognitive changes | 672 | 7.3 (1.7) | 4.5 (0.9) | 34.2 (10.9) | 7.3 (2.7) |
| Very much aware of negative cognitive changes | 177 | 6.9 (1.5) | 4.5 (0.8) | 32.5 (10.0) | 6.6. (3.0) |

N = number of participants. Not at all aware of negative cognitive changes = Score between 0 and 5 on the AARC-50 cognitive functioning subscale. A little aware of negative cognitive changes = Score between 6 and 10 on the AARC-50 cognitive functioning subscale. Moderately aware of negative cognitive changes = Score between 11 and 15 on the AARC-50 cognitive functioning subscale. Quite a bit aware of negative cognitive changes = Score between 16 and 20 on the AARC-50 cognitive functioning subscale. Very much aware of negative cognitive changes = Score between 21 and 25 on the AARC-50 cognitive functioning subscale.
